# A steep increase in healthcare seeking behaviour in the last months before colorectal cancer diagnosis

**DOI:** 10.1186/s12875-021-01482-0

**Published:** 2021-06-21

**Authors:** Josephina G. Kuiper, Myrthe P. P. van Herk-Sukel, Valery E. P. P. Lemmens, Ernst J. Kuipers, Ron M. C. Herings

**Affiliations:** 1 PHARMO Institute for Drug Outcomes Research, Van Deventerlaan 30-40, 3528 AE Utrecht, Netherlands; 2 Department of Public Health, Erasmus University Medical Center, Doctor Molewaterplein 40, 3015 GD Rotterdam, Netherlands; 3 Department of Internal Medicine and Dermatology, University Medical Center Utrecht, Heidelberglaan 100, 3584 CX Utrecht, Netherlands; 4 Netherlands Comprehensive Cancer Organisation (IKNL), Godebaldkwartier 419, 3511 DT Utrecht, Netherlands; 5 Department of Gastroenterology and Hepatology, Erasmus University Medical Center, Doctor Molewaterplein 40, 3015 GD Rotterdam, Netherlands; 6 Department of Epidemiology & Data Science, Amsterdam University Medical Center, De Boelelaan 1117, 1081 HV Amsterdam, Netherlands

**Keywords:** Primary healthcare, Colorectal cancer, Case–control, Netherlands

## Abstract

**Background:**

Timely recognition of colorectal cancer related symptoms is essential to reduce time to diagnosis. This study aims to investigate the primary healthcare use preceding a colorectal cancer diagnosis.

**Methods:**

From a cohort of linked cancer and primary care data, patients diagnosed with primary colorectal cancer in the period 2007–2014 were selected and matched to cancer-free controls on gender, birth year, GP practice and follow-up period. Primary healthcare use among colorectal cancer cases before diagnosis was compared with matched cancer-free controls. Mean monthly number of GP consultations and newly prescribed medication was assessed in the year before index date (diagnosis date for cases). Results were stratified by colorectal cancer site: proximal colon cancer, distal colon cancer and rectal cancer.

**Results:**

A total of 6,087 colorectal cancer cases could be matched to four cancer-free controls (*N* = 24,348). While mean monthly number of GP consultation were stable through the year among cancer-free controls, a statistical significant increase was seen among colorectal cancer cases in the last 4–8 months before diagnosis. Proximal colon cancer cases showed the longest time interval of increased mean monthly number of GP consultations. This increase was largely driven by a consultation for malignant neoplasm colon/rectum. The number patients receiving a newly prescribed medication was stable around 120 per 1,000 persons per month until 8 months before index date for proximal colon cancer cases, 4 months before index date for distal colon cancer cases and 3 months for rectal cancer cases. This increase was mainly driven by the prescription of laxatives drugs.

**Conclusion:**

An increase in the healthcare seeking behaviour of colorectal cancer patients prior to diagnosis was seen. The longest period of increased GP consultations and newly prescribed medication was seen among patients diagnosed with proximal colon cancer. This can be explained by the difficultly to diagnose proximal colon cancer given the more subtle signs compared to distal colon cancer and rectal cancer. Therefore, faster diagnosis for this specific tumour subtype may only be possible when clear clinical signs and symptoms are present.

## Background

The incidence of colorectal cancer continues to increase in Europe, with approximately 500,000 patients newly diagnosed with colorectal cancer each year [1]. Colorectal cancer is the second most common cause of cancer-related death in Europe, accounting for over 240,000 deaths each year.

In most European countries screening programmes have been implemented to improve outcomes and reverse the increasing incidence trend of colorectal cancer [2]. In the Netherlands, a national screening program was implemented in January 2014 with a participation rate of almost 75% in 2018 among people aged 55 to 75 years [3]. Despite population-based screening, patients remain to be diagnosed outside the context of screening either as interval cancer, due to non-participation or because they fall outside of the screening age range.

In the Netherlands, the general practitioner (GP) is the gatekeeper to specialist care, so it is likely that the GP is the first point of contact for people who experience health problems which may relate to cancer. Persistent rectal bleeding, blood in the stools, abdominal pain and bloating, loss of appetite and unexplained weight loss may all be signs of colorectal cancer and should at some point be a reason for further assessment and a referral for endoscopy [4]. Previous studies showed that the median time between first consultation with cancer-related complaints to referral varies greatly for colorectal cancer patients with duration of months and even years for 10–25% of the colorectal cancer patients [5, 6]. Delay in the diagnosis may have several important consequences, such as higher mortality and a more advanced disease stage. In order to achieve an earlier diagnosis of colorectal cancer, it is important to know the current healthcare seeking behaviour of colorectal cancer patients prior to diagnosis which may provide new knowledge on specific groups to refer for a diagnostic work-up.

We aimed to investigate the number of GP consultations among colorectal cancer patients and the medication they have been prescribed in the year before diagnosis.

## Methods

### Data sources

For this population-based case–control study, we used data from the Netherlands Cancer Registry (NCR) linked to the PHARMO GP Database (the NCR-PHARMO GP cohort). This cohort covers a catchment area of approximately 4 million inhabitants (approximately 20–25% of the Dutch population). The GP Database comprises data from electronic patient records registered by GPs including information on diagnoses and symptoms (coded according to the International Classification of Primary Care (ICPC) or entered as free text) and healthcare product/drug prescriptions (coded according to the World Health Organization (WHO) Anatomical Therapeutic Chemical (ATC) Classification System) [7].

The NCR is a population-based registry which is maintained by the Comprehensive Cancer Centre the Netherlands (IKNL) and comprises information on newly diagnosed cancer patients in the Netherlands. The NCR is notified for new patients with cancer by pathology departments, general hospitals, and radiotherapy institutes.

Further detailed information on the linkage and formation of the NCR-PHARMO GP cohort can be found elsewhere (Josephina G. Kuiper, Myrthe P.P. van Herk-Sukel, Valery E.P.P. Lemmens, Ernst J. Kuipers, Ron M.C. Herings: A population-based linked cohort of cancer and primary care data: a new source to study the management of cancer in primary care, submitted) [8].

### Study population

All patients who were diagnosed with primary colorectal cancer (International Classification of Diseases, Tenth Revisions, Clinical Modification (ICD 10-CM) code C18-C20) between January 1^st^ 2007 and December 31^st^ 2014 were selected. The first diagnosis date of colorectal cancer was defined as index date. Patients with a previous diagnosis of cancer (except basal cell skin carcinoma) were excluded. The same pertained to patients with less than 12 months of history available in the GP Database (defined as the time between the date of entering the PHARMO Database Network to the date of colorectal cancer diagnosis).

Each colorectal cancer case was matched to four cancer-free controls based on gender, birth year, GP practice and start follow-up in the PHARMO Database Network. Matched controls received the same index date as the diagnosis date in their matched case with colorectal cancer and could not be matched more than once. The same exclusion criteria for the cases were applied to the matched controls.

### Primary healthcare use

Information on primary healthcare use was derived from the GP Database of the PHARMO Database Network, which includes primary care data retrieved directly from the source, i.e. the electronic medical records of the healthcare providers.

According to the Medical Treatment Contract Act (“*Wet op de geneeskundige behandelingsovereenkomst*” (WGBO)), care providers (including general practitioners) are obliged to create and maintain a complete patient file for each patient. In the 1980s, the first automated system for general practices was introduced, replacing the handwritten files which were often difficult to read and incomplete. Since 2013, almost all general practices in the Netherlands work with an automated system to record the medical data of patients in the Electronic Patient File (EPD). In this EPD, GP consultations are grouped in episodes, i.e. a series of consultations related to a single reason for encounter (a symptom or a diagnosis). Besides information on the reason for encounter, prescriptions, laboratory results, referral letters and the summary of specialist letters are also registered in the system.

For this study, all GP consultations (face-to-face consultations, GP home visits and phone consultations) were extracted in the year prior index date for all colorectal cancer cases and their matched cancer-free controls. All information on diagnoses and symptoms registered by the GP (coded or entered as free text) was used for analyses. Furthermore, all prescribed medication based accompanied with an ATC codes in the year prior index date was extracted.

### Statistical analyses

Descriptive statistics were used to present baseline and tumour characteristics. The mean monthly number of GP consultations were calculated by dividing the monthly number of GP consultations by the number of colorectal cancer cases (or cancer-free controls) in each month. The Wilcoxon rank-sum test was used for comparing the mean monthly number of GP consultations between colorectal cancer cases and cancer-free controls. A *p*-value of < 0.05 was considered as a statistically significant difference.

Furthermore, in each month before the index date—going back 12 months—the number of colorectal cancer (or cancer-free controls) receiving a newly prescribed drug was assessed. Newly prescribed drugs were assessed based on the fourth level of the ATC code (chemical subgroup, i.e. A02BC) and defined as not receiving the drug in the year prior to that period. In each month, the incidence rate of patients receiving a newly prescribed medication was calculated by dividing the number patients receiving a newly prescribed medication by follow-up among colorectal cancer (or cancer-free controls) and presented per 1,000 persons per month. A Poisson regression analysis was used to examine whether the incidence rates per month significantly varied between colorectal cancer cases and cancer-free controls. A *p*-value of < 0.05 was considered as a statistically significant difference.

A *post-hoc analysis* was performed to assess the type of newly prescribed medication and reason for GP consultation that triggered the increase in the last 6 months before index date among colorectal cancer cases. The type of newly prescribed medication was assessed on the fourth level of the ATC code and presented if the absolute difference in receiving a specific drug between colorectal cancer cases and cancer-free controls was more than 5%. The reason for a GP consultation was determined by assessing the ICPC code associated with each consultation and by reviewing the free text of diagnosis and symptoms not accompanied by an ICPC code. Diagnoses and symptoms without an ICPC code (i.e. entered as free text) were reviewed and supplemented with an ICPC code were applicable. The reason for a GP consultation were separately listed based on ICPC codes if the absolute difference in the occurrence of a specific diagnosis or symptom between colorectal cancer cases and cancer-free controls was more than 2%.

As clinical features of colorectal cancer may vary significantly depending on the anatomical site, results were stratified by anatomical colorectal cancer site: proximal colon cancer, distal colon cancer and rectal cancer. Patients with colon cancer with an unspecified site or rectosigmoid cancer were not taken into account.

All data was analysed using SAS programs organized within SAS Enterprise Guide version 7.1 (SAS Institute Inc., Cary, NC, USA) and conducted under Windows using SAS version 9.4.

## Results

A total of 6,087 colorectal cancer cases could be matched to four cancer-free controls (*N* = 24,348) (Table 1). The mean (± SD) age of colorectal cancer cases and cancer-free controls was 68.7 (± 10.0) years and 56% was male. Of the colorectal cancer cases, 19% were diagnosed with stage I colorectal cancer, 27% with stage II, 31% with stage III and 20% with stage IV. For 3% the tumour stage at colorectal cancer diagnosis was unknown. The primary tumour was located in the distal colon in 33% of cases, 31% had a tumour located in the proximal colon and 32% in the rectum.

**Table 1:** Baseline characteristics of colorectal cancer cases and their matched cancer-free controls

**Characteristics**	**Colorectal cancer cases**	**Cancer-free control population**
	***N* = 6,087**	***N* = 24,348**
**Gender, n (%)**		
Male	3,434 (56)	13,736 (56)
Female	2,653 (44)	10,612 (44)
**Age at index date**		
< 44	79 (1)	316 (1)
45–54	493 (8)	1,972 (8)
55–64	1,405 (23)	5,620 (23)
65–74	2,208 (36)	8,832 (36)
75–84	1,652 (27)	6,608 (27)
≥ 85	250 (4)	1,000 (4)
Mean ± SD	68.7 ± 10.0	68.7 ± 10.0
**Year of diagnosis**		
2007–2009	1,495 (25)	5,980 (25)
2010–2012	2,451 (40)	9,804 (40)
2013–2014	2,141 (35)	8,564 (35)
**Duration of history available (years)**		
Mean (± SD)	4.7 ± 2.3	4.7 ± 2.3
**Tumour site**		
Colon	4,042 (66)	NA
Proximal	1,910 (31)	NA
Distal	2,022 (33)	NA
Unspecified	110 (2)	NA
Rectum	1,926 (32)	NA
Rectosigmoid	119 (2)	NA
**Tumour stage**		
I	1,171 (19)	NA
II	1,631 (27)	NA
III	1,884 (31)	NA
IV	1,242 (20)	NA
Unknown	159 (3)	NA

*SD* Standard deviation, *NA* Not applicable

**Fig. 1:**
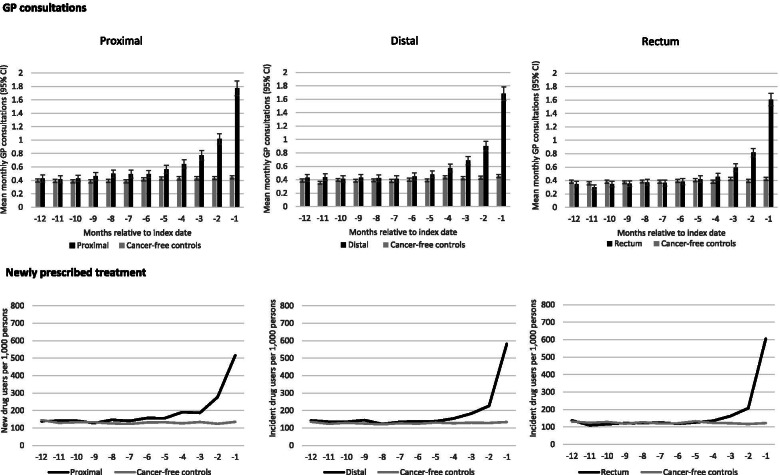
Mean monthly GP consultations and new drugs users in the year before index date, stratified by tumour site

For all different tumour sites, the mean monthly number of GP consultations increased in the last months before diagnosis, but the timing of the increased mean monthly number of GP consultations differed (Fig. 1). For proximal colon cancer, a statistically significant difference (*p*-value < 0.05) in GP consultation rates was observed from 8 months before diagnosis. This was 5 months for patients with distal colon cancer and 4 months in those with rectal cancer. The mean monthly GP consultation in the month before colorectal cancer diagnosis was highest among patients diagnosed with proximal colon cancer (1.8) compared with distal colon cancer (1.7) and rectal cancer (1.6).

The number of patients with newly prescribed medication was stable around 120 per 1,000 persons per month. A statistically significant difference (*p*-value < 0.05) in newly prescribed medication between colorectal cancer cases and cancer-free controls was seen from 8 months before index date for proximal colon cancer cases, 4 months before index date for distal colon cancer cases and 3 months for rectal cancer cases (Fig. 1). The highest number of patients with newly prescribed medication was seen among rectal cancer where it increased and peaked at 604 patients with newly prescribed medication per 1,000 persons in the month immediately before index date. The number of patients with newly prescribed medication among cancer-free controls remained stable throughout the year before index date.

In a post-hoc analysis assessing the type of newly prescribed drugs and reason for GP consultation that triggered the increase in the last months before diagnosed, it was seen that the increase in patients with newly prescribed medication was mainly driven by the prescription of laxatives drugs to help resolve constipation or empty the bowel before procedures or surgery involving the lower bowel, which were most often prescribed among patients diagnosed with rectal cancer (Table 2). Other drugs that were often newly prescribed included proton pump inhibitors, which were more often prescribed to patients diagnosed with proximal colon cancer (15 vs. 9% among patients diagnosed with distal colon cancer and 7% among patients diagnosed with rectal cancer). Drugs to treat iron deficiency anaemia were also a common newly prescribed drug especially among patients diagnosed with proximal colon cancer (14 vs. 4% among patients diagnosed with distal colon cancer and 2% among patients diagnosed with rectal cancer).

**Table 2:** Type of newly prescribed treatment and reason for GP consultation among colorectal cancer and matched cancer-free controls in the 6 months before index date, stratified by tumour site

	**Proximal**		**Distal**		**Rectum**	
	**Cases**	**Cancer-free controls**	**Cases**	**Cancer-free controls**	**Cases**	**Cancer-free controls**
	***N* = 1,910** **n (%)**	***N* = 7,640** **n (%)**	***N* = 2,022** **n (%)**	***N* = 8,088** **n (%)**	***N* = 1,926** **n (%)**	***N* = 7,704** **n (%)**
**Common newly prescribed medication**						
**A06AD**						
*Osmotically acting laxatives*	729 (38)	175 (2)	928 (46)	194 (2)	914 (48)	173 (2)
**A06AB**						
*Contact laxatives*	260 (14)	32 (< 0.5)	426 (21)	47 (1)	444 (23)	36 (1)
**A02BC**						
*Proton pump inhibitors*	288 (15)	373 (5)	182 (9)	377 (5)	143 (7)	337 (4)
**B03AA**						
*Iron bivalent, oral preparations*	273 (14)	32 (< 0.5)	71 (4)	42 (1)	31 (2)	25 (< 0.5)
**A06AG**						
*Enemas*	40 (2)	22 (< 0.5)	117 (6)	35 (< 0.5)	50 (3)	23 (< 0.5)
**M01AB**						
*Acetic acid derivatives and related substances*	116 (6)	303 (4)	106 (5)	300 (4)	76 (4)	323 (4)
**Common reason for GP consultation**						
**D75**						
*Malignant neoplasm colon/rectum*	664 (35)	1 (< 0.5)	780 (39)	2 (< 0.5)	860 (45)	0 (0)
**D16**						
*Rectal bleeding*	10 (1)	13 (< 0.5)	94 (5)	11 (< 0.5)	78 (4)	12 (< 0.5)
**B80**						
*Iron deficiency anaemia*	99 (5)	9 (< 0.5)	27 (1)	10 (< 0.5)	15 (1)	2 (< 0.5)
**B82**						
*Anaemia other/unspecified*	64 (3)	11 (< 0.5)	12 (1)	10 (< 0.5)	9 (1)	12 (< 0.5)
**D06**						
*Abdominal pain localized other*	44 (2)	21 (< 0.5)	41 (2)	37 (1)	9 (1)	14 (< 0.5)

The increase in monthly GP consultations in the 4–9 months before index date was largely driven by a consultation for malignant neoplasm colon/rectum. Consultations for rectal bleeding was more prominent among distal colon cancer and rectal cancer compared to proximal colon cancer (5 and 4% versus 1%, respectively). Patients diagnosed with proximal colon cancer more often had a GP recording for iron deficiency anaemia or other anaemia compared to patients diagnosed with distal colon cancer or rectal cancer.

## Discussion

An increase in the mean monthly number of GP consultations and patients with newly prescribed medication was seen in the year before colorectal cancer diagnosis compared to a cancer-free control population with a steep increase in the last months before diagnosis. This increase was seen for all anatomic sites of colorectal cancer, but the timing of the increase differed. Patients diagnosed with proximal colon cancer had the longest period with increased GP consultations rates and newly prescribed drugs compared to cancer-free controls.

The increase in GP consultations rate in the months before colorectal cancer diagnosis was mainly driven by a contact coded as malignant neoplasm of colon/rectum. This might indicate that there is a small difference in the date of diagnosis recorded by the GP and the NCR. A previous study assessing the quality of cancer registration in Dutch primacy care showed that in 80.6% of the cases the year of diagnosis in the primary care electronic health records is registered in accordance with the NCR [9]. For the cases with a different recorded year of diagnosis, the deviation was found to be less than two years. It could also be that the GP already recorded a diagnosis of colorectal cancer based on certain definite symptoms or results from the faecal immunochemical tests without a confirmation from a specialist. Other reasons for the increase in consultations rates were rectal bleeding, iron deficiency anaemia or other, and abdominal pain. These are known alarm symptoms for colorectal cancer and especially rectal bleeding and anaemia warrant further investigation, irrespective of whether other symptoms are present [4].

Tumours arising from the proximal colon tend to present with more subtle signs such as anaemia compared to tumours arising from the distal colon [10]. This was also seen in our study in which proximal colon cancer cases presented more often with anaemia compared to distal colon cancer and rectal cancer cases. The more subtle signs of proximal colon cancer also explain the slightly longer increased intervals of GP consultations and newly prescribed drugs among proximal colon cancer as these cancers are more difficult to diagnose compared to distal colon cancer and rectal cancer. Although we know that there are differences between anatomic site of colorectal cancer in terms of developmental origin and molecular and genetic characteristics, there was only one previous study found that assessed differences in GP consultation rates and prescriptions between different tumour locations [11]. Similar as in our study, this study also showed that patients diagnosed with proximal colon cancer had the longest intervals with increased rates of GP consultations. Rectal cancer patients had long intervals with higher prescription rates than references. This was not seen in our study, but we determined newly prescribed drugs in contrast to a previous study that only took prescriptions for haemorrhoids into account. These differences indicates that each anatomic site of colorectal cancer should be considered separately and have a different presentation in primary care.

Other previous research also showed an increased GP consultation rate before colorectal cancer diagnosis [11–15], but only a few studies also looked at the reasons for and the contents of the consultations with the GPs. In a previous study in which also data from Dutch general practices were used but a different region, the largest difference was observed for contacts related to the digestive system (coded as ICPC-D): 46.0% of patients with colorectal cancer showed two or more contacts for these reasons in the year before diagnosis, compared with 12.2% of controls. This specific ICPC chapter also includes the code related to malignant neoplasm of the colon/rectum and might also be in this study the main reason for the increase as index date was defined as a referral to colonoscopy indicating that the GP might already suspect colorectal cancer [15].

Laxatives drugs were often newly prescribed in the 6 months before diagnosis, most likely as preparation of a colonoscopy to clear the upper bowel. Similar to the prescription of enema to empty the lower part of the bowel, but less often prescribed compared to laxatives. An increase in prescribed drugs before colorectal cancer diagnosis was also seen in other previous studies with a peak in the last month before colorectal cancer diagnosis. In line with our study, drugs used for constipation showed the highest increase in use [16, 17]. Proton pump inhibitors were also common newly prescribed, especially among patients diagnosed with proximal colon cancer. Tumours in the proximal colon may result in symptoms that are similar to diseases in the upper gastrointestinal tract, such as the stomach. It is unlikely that proton pump inhibitors are prescribed to treat colorectal cancer if they expect the pain to be cancer related.

A strength of this study is the use of a database with GP recorded information extracted directly from the source instead of survey data which may lead to inaccurate information on primary care use. In the Netherlands, every inhabitant is registered with a GP which allows comprehensive follow-up of patients in the primary care setting. Thereby, information on the actual diagnosis of colorectal cancer was obtained from the NCR. As shown in a previous study, 40% of cancer cases can be missed when using only GP recorded information, and almost half can be false positive [9]. Relying solely on GP recorded information will result in misclassification of colorectal cancer cases and cancer-free controls and will bias the results. We observed some GP recorded diagnosis of colorectal cancer among cancer-free controls, which was not recorded in the NCR. This may indicates a false positive diagnosis potentially as a result of incorrectly using a diagnostic coding for coding symptoms as actual cancer. Given the very low number of cancer-free controls with a diagnosis code for colorectal cancer in primary care data we do not expect that this affected the results.

GPs are obligated to record all relevant information about the patient in their medical files, but are not obliged to code all diagnoses and symptoms with an ICPC code. In this study, besides coded diagnosis and symptoms also uncoded diagnoses and symptoms entered as free text were identified and included. As uncoded diagnosis and symptoms were manually reviewed and supplemented with an ICPC code where applicable, some signs of colorectal cancer may have been missed. However, as only less than 10% of the information recorded by the GP was uncoded, we expect that we have missed only a few or none uncoded signs of colorectal cancer and did not affect the results. Furthermore, colorectal cancer cases were matched – among other things – with cancer-free controls on GP practice resulting in evenly distributed inaccuracies in recording and prescribing among colorectal cancer cases and cancer-free controls.

## Conclusions

In conclusion, this large population-based study showed a steep increase in the number of GP consultations and patients with newly prescribed medication especially in the last 4–8 months before colorectal cancer diagnosis. This increased healthcare use can be seen as a proxy variable for symptom presentation. The longest period of increased primary healthcare use was seen among patients diagnosed with proximal colon cancer, which may indicate a potential for a faster diagnostic pathway among this specific tumour subtype. The differences in the period of increased primary healthcare use between anatomic site can be explained by the difficulty to diagnose proximal colon cancer compared to distal colon cancer and rectal cancer. Shortening the diagnostic pathway may therefore only be possible among those patients presenting in primary care with clear signs and symptoms.

## Data Availability

The datasets generated and analysed during the current study are not publicly available due to privacy reasons but are available from the corresponding author on reasonable request.

## Abbreviations

ATC: Anatomical Therapeutic Chemical
CM: Clinical Modification
EPD: Electronic Patient File
GP: General practitioner
NCR: Netherlands Cancer Registry
ICD: International Classification of Diseases
ICPC: International Classification of Primary Care
IKNL: Comprehensive Cancer Centre the Netherlands
SD: Standard deviation
STIZON: Stichting Informatievoorziening voor Zorg en Onderzoek
WGBO: Wet op de geneeskundige behandelingsovereenkomst
WHO: World Health Organization

## References

[1] Ferlay J, Colombet M, Soerjomataram I, Dyba T, Randi G, Bettio M (2018). Cancer incidence and mortality patterns in Europe: estimates for 40 countries and 25 major cancers in 2018. Eur J Cancer.

[2] Schreuders EH, Ruco A, Rabeneck L, Schoen RE, Sung JJ, Young GP (2015). Colorectal cancer screening: a global overview of existing programmes. Gut.

[3] Toes-Zoutendijk E, Portillo I, Hoeck S, de Brabander I, Perrin P, Dubois C (2020). Participation in faecal immunochemical testing-based colorectal cancer screening programmes in the northwest of Europe. J Med Screen.

[4] Astin M, Griffin T, Neal RD, Rose P, Hamilton W (2011). The diagnostic value of symptoms for colorectal cancer in primary care: a systematic review. Br J Gen Pract.

[5] van Erp NF, Helsper CW, Olyhoek SM, Janssen RRT, Winsveen A, Peeters PHM (2019). Potential for reducing time to referral for colorectal cancer patients in primary care. Ann Fam Med.

[6] Helsper CCW, van Erp NNF, Peeters P, de Wit NNJ (2017). Time to diagnosis and treatment for cancer patients in the Netherlands: room for improvement?. Eur J Cancer.

[7] WHO Anatomical Therapeutic Chemical Classification System. www.whocc.no/atc_ddd_index. Accessed 7 May 2021.

[8] Kuiper JG, van Herk-Sukel MP, Lemmens VEPP, van Wijngaarden R, Herings RCM (2017). Insight into the role of the general practitioner in the management of colorectal cancer: record linkage of the Netherlands Cancer Registry and the General Practitioner Database of the Pharmo Database Network. Value Health.

[9] Sollie A, Roskam J, Sijmons RH, Numans ME, Helsper CW (2016). Do GPs know their patients with cancer? Assessing the quality of cancer registration in Dutch primary care: a cross-sectional validation study. BMJ Open.

[10] Sideris M, Adams K, Moorhead J, Diaz-Cano S, Bjarnason I, Papagrigoriadis S (2015). BRAF V600E mutation in colorectal cancer is associated with right-sided tumours and iron deficiency anaemia. Anticancer Res.

[11] Hansen PL, Hjertholm P, Vedsted P (2015). Increased diagnostic activity in general practice during the year preceding colorectal cancer diagnosis. Int J Cancer.

[12] Morrell S, Young J, Roder D (2019). The burden of cancer on primary and secondary health care services before and after cancer diagnosis in New South Wales, Australia. BMC Health Serv Res.

[13] Ewing M, Naredi P, Nemes S, Zhang C, Mansson J (2016). Increased consultation frequency in primary care, a risk marker for cancer: a case-control study. Scand J Prim Health Care.

[14] Jensen H, Vedsted P, Moller H (2018). Consultation frequency in general practice before cancer diagnosis in relation to the patient’s usual consultation pattern: a population-based study. Cancer Epidemiol.

[15] Brandenbarg D, Groenhof F, Siewers IM, van der Voort A, Walter FM, Berendsen AJ (2018). Possible missed opportunities for diagnosing colorectal cancer in Dutch primary care: a multimethods approach. Br J Gen Pract.

[16] Pottegard A, Hallas J (2017). New use of prescription drugs prior to a cancer diagnosis. Pharmacoepidemiol Drug Saf.

[17] van Erning FN, Zanders MM, Kuiper JG, van Herk-Sukel MP, Maas HA, Vingerhoets RW (2016). Drug dispensings among elderly in the year before colon cancer diagnosis versus matched cancer-free controls. J Clin Pharm Ther.

